# Everybody's Page

**Published:** 1888-10-13

**Authors:** 


### Everybody's Page.

Cocoa-nut oil is used for the manufacture of marine soaps, because, unlike all other kinds, it is not rendered insoluble by brine, and therefore will produce lather when used with sea water.

According to Dr. Harrington, of the Harvard Medical School, the diabetic foods commonly sold contain but a trifle less starch than ordinary bread, and he points out the danger which attends their use by persons suffering from diabetes, and who accept as true that these foods are non-starchy.

Quill toothpicks are coming in large quantities from France. The largest factory in the world is near Paris, where there is an annual product of 20,000,000 quills. The factory was started to make quill pens, but when these went out of use it was turned into a toothpick mill.

Women should especially note that the garter is objectionable, as causing a congestion of the blood, which, even without this accessory, requires to be guarded against under certain conditions. It is better to wear long stockings, and to keep them in position by suspenders.

Some drugs, notably salicin and quinine, when used as internal remedies, are productive of singing in the ears and deafness. The latter in large doses induces marked congestion of the auditory apparatus, and thus, if long administered, may cause disease of the labyrinth and internal ear, and resultant incurable deafness.

It is announced that paper bottles are to be manufactured on a very extensive scale; their weight is less than those of glass or stoneware, and they are less liable to breakage. Paper being also an excellent non-conductor, fluids stored in air-tight paper bottles will withstand a more intense degree of heat or cold than when put in ordinary bottles.

The Massachusetts Legislature has been wrestling with the subject of labelling and restricting the sale of poisons. Among other things, the red label has been adopted for certain drugs. Bills are also pending that have reference to the fee for licensing drug stores, the registering of alcohol, and the exemption of druggists from Jury duty.

Sulphate of quinidine, at one time almost a drug in the market, at other times in considerable demand, and justly esteemed as being practically equivalent to quinine in therapeutic value, is becoming more and more scarce, because manufacturers of quinine at the present time use Javanese or East Indian barks almost exclusively, and these contain either no quinidine at all, or so little of it, that its separation does not pay.

The serious results of lace-work, in which the eyes are severely taxed, compelling nearly all who work at it to use magnifying glasses and leading to premature decay of the eyes and loss of vision, and the impairment of vision complained of by girls who work at night on articles of dress of a black colour, are sufficient evidence of the injurious effects of sewing if too fine or prolonged, or performed under unfavourable conditions of light.

A new cement for bottles, containing very volatile liquids, which is easily prepared and applied, and which is said to prevent the escape of the most volatile liquids, is composed simply of very finely-ground litharge and glycerine, and it is merely painted around the joint between the bottle and the cork or stopper. It quickly dries and becomes extremely hard, but can be easily scraped off with a knife when it is necessary to open the bottle.

In the progress of Christianity, the elegance of long hair being deemed inconsistent with the profession of persons bearing the cross, it was prohibited by numerous canons and injunctions, the clergy of the Eastern Churches discountenancing the practice by their own examples. All such measures, however, proving ineffectual, a canon was promulgated in 1096 stating that such as wore long hair should be excommunicated while living and remain unprayed for when dead. In the present day the Greek and Russian clergy allow their hair to grow as it likes.

A Dundee newspaper, which holds weekly prize competitions, offered lately the usual prize of a silver watch for the most nonsensical verse that their competitors could compose. The following was the successful squib, and it possesses a melancholy interest for chemists:—

There was a mad druggist of Warwick,  
Who invented a new paregoric;  
When he found 'twouldn't sell, he set fire to his well,  
And went and got drowned in a straw-rick!

It is reported that in Peru and other parts of South America this year's fruit has been avoided by birds, while it has caused the death of sheep and cattle when fed to them in large quantities. These observations have been cited as tending to show that the instinct of birds with respect to the wholesomeness of fruits is frequently a worthy guide for human beings to follow. The possibility is suggested that the variation in the fruit of different years may have something to do with outbreaks of cholera.

The rationale of the cleansing action of soap is as follows: The dirt adhering to the body is mostly composed of dust and other extraneous matter combined with the perspiration and the greasy excretion from the skin. When ordinary hard soap is brought into contact with water it is decomposed and gives up part of its soda, which unites with and removes from the skin the unctuous dirt thus rendered miscible in the water used for washing. As the more the liberated soda is diluted the less will be its caustic effect on the skin, it is clear that a liberal supply of water should accompany the use of soap in personal ablution.

Paul Noirot, of Paris, is the patentee of a new method of preparing extract of coffee which is said to yield a very satisfactory product. Roasted and ground coffee of the best quality is treated with boiling water in the usual manner, and the infusion caused to freeze by appropriate refrigerating apparatus. The ice crystals are rapidly crushed and separated in a centrifugal machine from the dense accompanying extract, which does not itself freeze. This extract, which is thus freed from about ninety per cent. of its aqueous constituent, is then completely deprived of water in a vacuum apparatus, and the residue made into tablets or cakes.

The comparative freedom of the English race from myopia may be regarded as being due in great measure to the habits of their ancestors for many generations past. Their Danish and Saxon forefathers lived on the sea or in the open air, where distant objects attracted notice, while manufactures were few in number, and of workmanship involving close examination there was little or none. The imperfection of the means of lighting at night, the absence of books, and the ignorance of letters, led to drinking and story-telling, but led also to early rest, long sleep, and to a healthy form and construction of the eyes. It is only the strong development of studious tastes among our youth of late years that has rendered short-sightedness so common that we hardly think a scientific or literary man or woman complete without spectacles, or at least a *pince-nez*

